# Structured reporting enhances diagnostic quality in periapical dental radiographs: a comparative evaluation

**DOI:** 10.3389/fdmed.2025.1695707

**Published:** 2026-01-07

**Authors:** Moritz Ludwig Schnitzer, Anna-Lisa Forster, Gloria Biechele, Felix L. Herr, Christian Dascalescu, Maurice Heimer, Ricarda Ebner, Viktoria Fusch, Matthias Frank Frölich, Tobias Graf, Johannes Rübenthaler, Thomas Geyer

**Affiliations:** 1 Department of Radiology, University Hospital Munich, Ludwig Maximilian University of Munich, Munich, Germany; 2 Department of Radiology and Nuclear Medicine, University Medical Center Mannheim, Heidelberg University, Mannheim, Germany; 3 Department of Prosthodontics, Center for Dentistry and Oral Medicine (Carolinum), Goethe University, Frankfurt Am Main, Germany

**Keywords:** structured reporting, periapical radiographs, dental diagnostics, radiographicInterpretation, clinical documentation, free-Text reports

## Abstract

**Background:**

Radiological reports are critical for accurate diagnosis and therapeutic decision-making. While narrative free-text reports remain the conventional standard in dental radiology, structured reporting has emerged as a promising approach to enhance report quality, consistency, and clinical relevance. This study aims to assess whether structured reporting provides measurable advantages over traditional narrative reports in the interpretation of dental radiographs.

**Materials and methods:**

A total of 50 randomly selected narrative reports of intraoral dental radiographs were retrospectively analyzed. Using a standardized template, corresponding structured reports were created for each case. Two independent dentists evaluated the reports using a detailed questionnaire, comparing both formats across nine parameters: therapeutic decision-making, completeness, information extraction, level of detail, logical sequence, trustworthiness, linguistic quality, clarity, and overall assessment.

**Results:**

Structured reports showed significantly higher ratings in terms of completeness, information extraction, detail, trust, linguistic quality, clarity, and overall evaluation (*p* < 0.001). No significant differences were observed between structured and narrative reports regarding therapeutic decision-making or the sufficiency of information for treatment planning.

**Conclusion:**

Structured reporting in dental radiology demonstrates clear benefits in report clarity, quality, and interpretive utility. Although its impact on clinical decision-making may be equivalent to narrative reports, its consistent structure offers valuable advantages for communication, documentation, and future integration with clinical decision support systems.

## 1 Introduction

Intraoral radiography remains one of the most fundamental diagnostic tools in contemporary dental practice. Periapical radiographs, in particular, provide high-resolution imaging of individual teeth and their surrounding structures, enabling detailed evaluation of root morphology, periapical lesions, bone levels, endodontic status, and dental restorations. These images serve as a cornerstone in the detection of pathologies such as apical periodontitis, root fractures, and carious lesions that are often invisible during clinical examination alone (1–3).

Due to their diagnostic precision, low radiation exposure, and relative ease of acquisition, periapical radiographs are routinely employed in both general dentistry and specialist disciplines, including endodontics, periodontology, and oral surgery (1, 4, 5). The quality and clarity of the radiographic image, however, must be complemented by a well-structured and clinically meaningful report to ensure that the findings are accurately interpreted and effectively communicated. Radiological interpretation not only influences treatment decisions, but also plays a vital role in documentation, medico-legal clarity, and interdisciplinary communication (6–8).

Traditionally, dental radiographic findings have been documented in free-text form, allowing clinicians to describe observations with narrative flexibility. While this approach offers freedom in expression, it is also subject to inconsistency, omission of key findings, and limited standardization—particularly when used across different practitioners or institutions. These limitations have prompted growing interest in structured reporting (SR), a method that organizes radiological observations into predefined categories, often supported by standardized terminology and decision trees (9–13).

In medicine, structured reporting has been shown to improve the clarity, completeness, and reproducibility of radiological documentation (10, 14–17). In dental radiology, however, its implementation remains limited. Given the increasing demands for quality assurance, digital documentation, and data-driven diagnostics, structured reporting may represent a timely and necessary evolution in the field.

This study aims to evaluate the benefits and limitations of structured reporting for dental periapical radiographs in comparison to traditional narrative reports, with a focus on diagnostic completeness, readability, and clinical utility.

## 2 Materials and methods

### 2.1 Study design

This clinical retrospective study was conducted at the Department of Radiology, University Hospital Innenstadt, Ludwig-Maximilians-University of Munich (LMU). The study involved the analysis of 50 randomly selected narrative reports (NRs) based on retrospectively available, anonymized dental radiographs.

A structured reporting template specifically tailored for dental radiographs was created using the online platform *Smart Radiology*. Based on this template, structured reports were generated for each of the 50 previously selected cases.

The primary objective of the study was to evaluate whether structured reporting could offer measurable advantages over traditional narrative reporting in the interpretation and documentation of dental radiographs.

To compare SRs with NRs, a custom-designed questionnaire was developed to systematically assess report quality. This questionnaire was completed independently by two experienced dentists. The collected responses were then analyzed to identify potential differences between the two reporting formats.

The study received ethical approval from the Ethics Committee of the Medical Faculty, LMU Munich (Reference: 23-0172; Date of Approval: February 24, 2023). Data collection was performed in an anonymized manner, and the study adhered to the ethical standards outlined in the Declaration of Helsinki.

### 2.2 Data collection and acquisition

#### 2.2.1 Image acquisition

All retrospectively available radiographic images were acquired using a *Heliodent Plus* x-ray unit (Dentsply Sirona) in combination with a digital intraoral sensor. To ensure consistently high image quality, all dental radiographs were obtained using the paralleling technique with standardized positioning aids, including sensor holders, bite blocks, and alignment rings.

The selection of 50 dental radiographs was performed randomly from a non-selective, uncontrolled patient cohort, without applying any inclusion or exclusion criteria beyond image availability and anonymization.

#### 2.2.2 Generation of the reporting template

To conduct this clinical retrospective study, the development of a suitable reporting template specifically tailored to the structured reporting of dental radiographs was essential. For this purpose, the *Smart Radiology* platform by Smart Reporting GmbH (Munich, Germany) was utilized. This web-based tool offers users access to pre-defined structured reporting templates known as *smart templates*. Additionally, users can adapt existing templates or create fully customized versions using an integrated template editor.

For this study, a dedicated template titled ‘Dental Radiographs’ was developed. The template is divided into four primary sections: Procedure, Clinical Information, Prior Examinations, and Findings. Each section contains various subcategories, which can be activated via mouse click. The user is guided through a structured decision tree composed of yes/no questions, free-text fields, and single- or multiple-choice options. As selections are made, predefined text blocks are automatically generated to create a complete and coherent final report. Once all relevant items are selected, the structured report is automatically generated and can be exported seamlessly for integration into the dental clinic's documentation system.

#### 2.2.3 Generation of free-text reports

The sample size of 50 paired cases was determined in line with comparable, published studies on structured reporting in other radiological disciplines to provide an adequate basis for the comparative quality assessment (18–22). The 50 narrative reports used for comparison in this study were produced during routine clinical practice by three independently practicing, licensed dentists. These practitioners had 10–15 years of clinical experience in private practice in Germany, with special interests including Periodontology, Endodontology, Prophylaxis, and Implantology. These reports represented the conventional standard for daily diagnostic documentation and were inherently used by the practitioners as the basis for clinical decision-making and treatment planning in the respective patient cases. These reports were created without the use of text modules or predefined templates, ensuring that each was composed freely and individually. The dental radiographs and their corresponding reports were randomly selected and anonymized prior to export.

#### 2.2.4 Generation of structured reports

**Figure 1:**
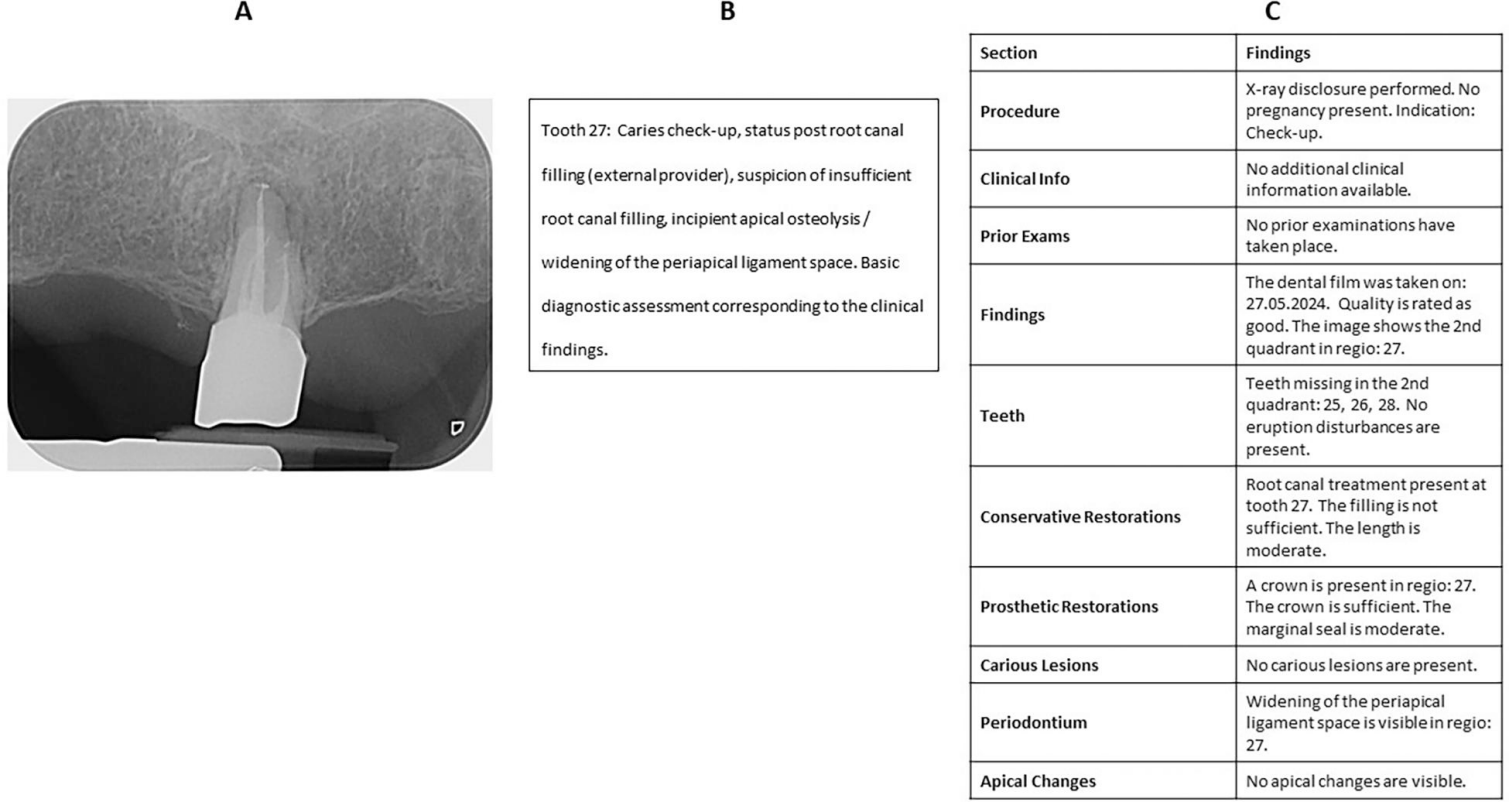
Example radiograph with narrative and structured report. **(A)** Representative periapical radiograph of tooth 27. **(B)** Narrative free-text report as originally produced in routine clinical practice. **(C)** Structured report generated with the dedicated template used in this study, showing the predefined sections (procedure, clinical information, prior exams, findings, teeth, restorations, periodontal findings, apical changes).

For all 50 dental radiographs included in the study, structured reports were retrospectively created by a licensed dentist. By systematically selecting appropriate options within the decision tree and, where necessary, adding supplemental free-text input, all relevant fields within the template were completed. Based on these inputs, the system generated a fully structured report using predefined text modules. The finalized structured reports were then exported via the platform's export function and saved in Microsoft Word format for further evaluation. A representative periapical radiograph with its corresponding narrative report and structured report is provided in Figure 1 to illustrate the qualitative differences between the two documentation formats.

#### 2.2.5 Evaluation of reports

**Figure 2:**
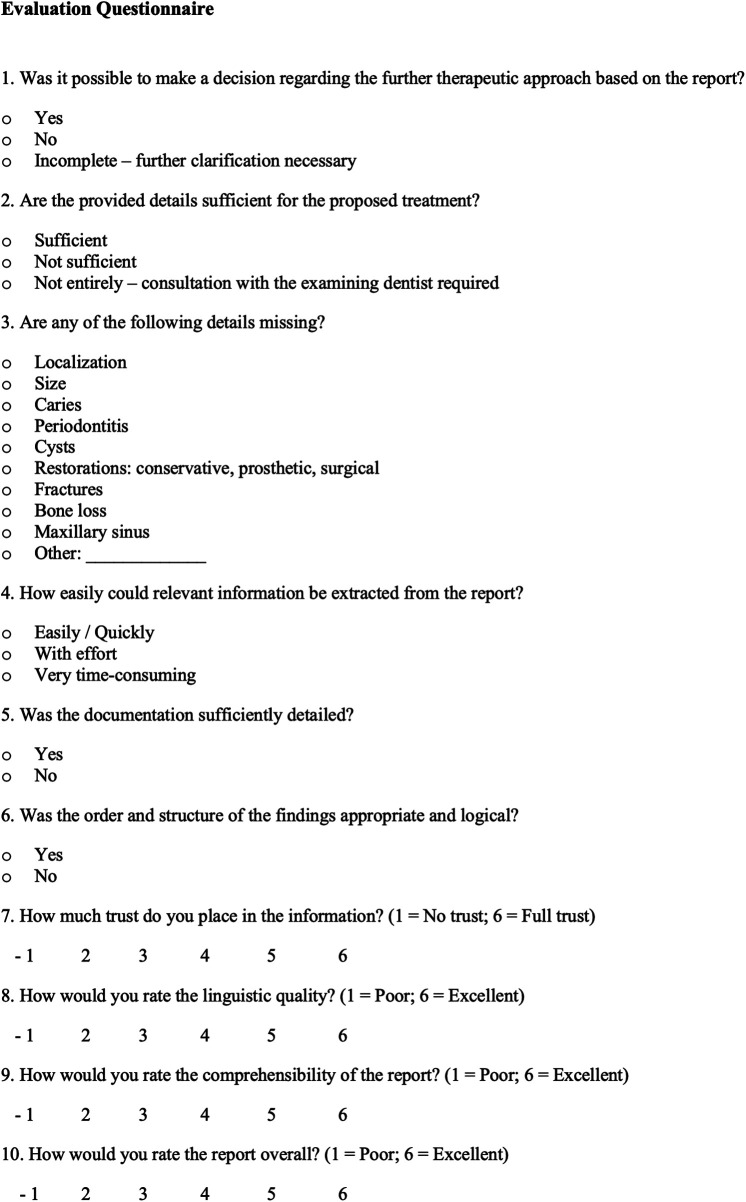
Questionnaire for the evaluation of NR and SR of dental radiographs evaluated by two independent dentists.

All structured and narrative reports included in this investigation were independently assessed by two licensed dentists. These evaluators both had 10–15 years of extensive clinical experience in private practice in Germany, with special interests in Periodontology, Endodontology, Prophylaxis, and Implantology. The evaluation employed a purpose-built questionnaire developed specifically for this study to measure report quality and practical relevance in the context of dental radiography (Figure 2). The evaluation instrument was developed by the same clinican who designed the structured reporting template based on established literature criteria for radiological report quality (e.g., completeness, logical structure, and suitability for clinical use). To ensure its validity, the questionnaire underwent rigorous internal content validity assessment and refinement by the entire author team, including senior clinical and radiological experts, before being finalized for use by the independent evaluators.

The assessment focused on several core aspects: whether the clinician's diagnostic question was adequately addressed; whether the report contained sufficient information to support clinical decision-making and treatment planning; and whether the documented findings derived from the periapical radiographs were appropriate for the suggested therapeutic approach. Additionally, reviewers recorded any omissions of key diagnostic content. Furthermore, the reviewers rated the ease of information retrieval, the logical structure of the report, and the degree of trust they placed in its content. Linguistic clarity and overall report quality were evaluated using a six-point Likert scale (1 = insufficient; 6 = excellent).

To ensure the consistent application of the evaluation instrument, the key subjective constructs were operationally defined for the evaluators as follows: Trust was defined as the evaluator's level of subjective confidence in the accuracy of the report's conclusion and therapeutic recommendation. Logical Structure assessed the systematic organization and clinically intuitive flow of the reported findings. Finally, Ease of Information Retrieval measured the efficiency and speed with which a critical piece of data could be accurately located and extracted from the documentation.

To reduce potential bias, all reports underwent a two-step preparation process. First, anonymization was ensured by meticulously removing all patient and practitioner identifiers, dates, and practice details from both report formats. Second, randomization was performed to control for sequence and recall bias: the 100 reports (50 NR and 50 SR) were presented to the two independent evaluators in a fully randomized and interleaved sequence. Reports were individually paired with their corresponding radiographic images. This pairing was essential, as key evaluation constructs, such as ‘appropriateness’ and ‘trust,’ required the reviewers to assess the report's fidelity and sufficiency against the visual findings of the corresponding radiograph. Furthermore, to maintain a high level of rigor, the assessment was performed by both experienced evaluators using standardized professional computer equipment under routine clinical viewing conditions. A minimum separation of two weeks was maintained between the assessment of the two reports belonging to the same case by the same evaluator. Questionnaires were completed immediately following each assessment to preserve the integrity and objectivity of the responses.

### 2.3 Statistical methods

The statistical evaluation of the collected data was conducted using *IBM SPSS Statistics*, version 29.0.2.0 (IBM Corp., Armonk, NY, USA). The primary aim of the analysis was to assess differences in the evaluation of NRs and SRs, and to determine whether either reporting method demonstrated a statistically significant advantage. All aspects assessed in the questionnaire—including completeness, clarity, and linguistic quality—were incorporated into the analysis. The significance level for all tests was set at *α* = 0.05.

The questionnaire consisted of various item formats, including dichotomous (Yes/No), ordinal-scaled (e.g., 1–6 rating), multiple-choice, and open-text responses, requiring the application of different statistical procedures. Descriptive statistics were used to calculate frequencies for nominal variables, which were visualized using bar charts. For ordinal-scaled variables, descriptive measures including means, medians, modes, standard deviations, ranges, and percentiles were computed separately for both report types (NRs and SRs).

To compare two dependent, paired groups, the McNemar test was applied. For ordinal-scaled the Wilcoxon signed-rank test was employed. A *p*-value less than 0.05 was considered indicative of a statistically significant difference in perceived report quality. In addition, inter-rater agreement between the two dentists was assessed using Cohen's Kappa and Krippendorff's Alpha. To incorporate the multiple-choice responses and the open-ended “Other”-option, frequencies and percentages of missing information were analyzed and visualized in a separate chart. Free-text responses were subsequently analyzed qualitatively and interpreted in a dedicated section.

## 3 Results

### 3.1 Therapeutic decision-making

In 98% of NRs and 99% of structured reports SRs, sufficient information was available to make a therapeutic decision. Only 1% of cases in both formats required further clarification. No significant difference was observed between the two reporting methods in this regard.

### 3.2 Sufficiency of information for treatment

Both narrative and structured reports were rated 100% sufficient for supporting treatment decisions. Median and mode values were identical (1.00), indicating no difference between the two formats in terms of informational adequacy.

### 3.3 Missing information

Narrative reports lacked essential information in 91% of cases, while structured reports were rated as complete in 96% (*p* < 0.001). Major gaps in NRs included dental restorations, bone loss, and periodontal findings. Frequent additions in free-text fields involved missing teeth, root filling quality, and third molar assessments. Structured reports showed significantly higher completeness across nearly all categories.

### 3.4 Information extraction

Relevant information was rated as “easy to extract” in 91% of structured reports, compared to 72% of narrative reports. Only SRs avoided being rated as “very time-consuming.” In contrast, 7% of NRs received this rating. These findings suggest that SRs offer clearer structure and better accessibility of key information.

### 3.5 Level of detail in documentation

Structured reports were rated as sufficiently detailed in 98% of cases, compared to only 53% for narrative reports (*p* < 0.001). Nearly half of the NRs were considered lacking in detail, whereas SRs showed consistently high ratings, indicating a significantly greater level of documentation precision.

### 3.6 Logical sequence of report structure

While only 54% of narrative reports were rated as logically structured, 100% of structured reports were considered coherent and appropriately ordered. This significant difference highlights the superior clarity and standardization of SRs.

### 3.7 Diagnostic confidence

Structured reports achieved significantly higher trust ratings, with 96% of responses indicating ‘full confidence’ and 4% ‘high confidence.’ In contrast, narrative reports showed greater variability, with only 11% receiving the top score and 30% rated as low to no confidence. Mean confidence was 5.96 for SRs vs. 4.16 for NRs (*p* < 0.001), indicating markedly greater trust in structured documentation.

### 3.8 Linguistic quality

Structured reports were rated as ‘excellent’ in 98% of cases and ‘good’ in the remaining 2%. None of the narrative reports reached the top score; 50% were rated as ‘good,’ while the rest varied widely. SRs showed significantly higher and more consistent language quality (mean 5.98 vs. 3.90; *p* < 0.001).

### 3.9 Comprehensibility

Structured reports were rated as ‘excellent’ in 95% and ‘good’ in 5% of cases, showing minimal variance. Narrative reports varied widely, with only 1% rated as ‘excellent’ and a mean score of 3.71 vs. 5.95 for SRs (*p* < 0.001). These results highlight the significantly higher clarity of SRs.

### 3.10 Overall assessment

**Figure 3:**
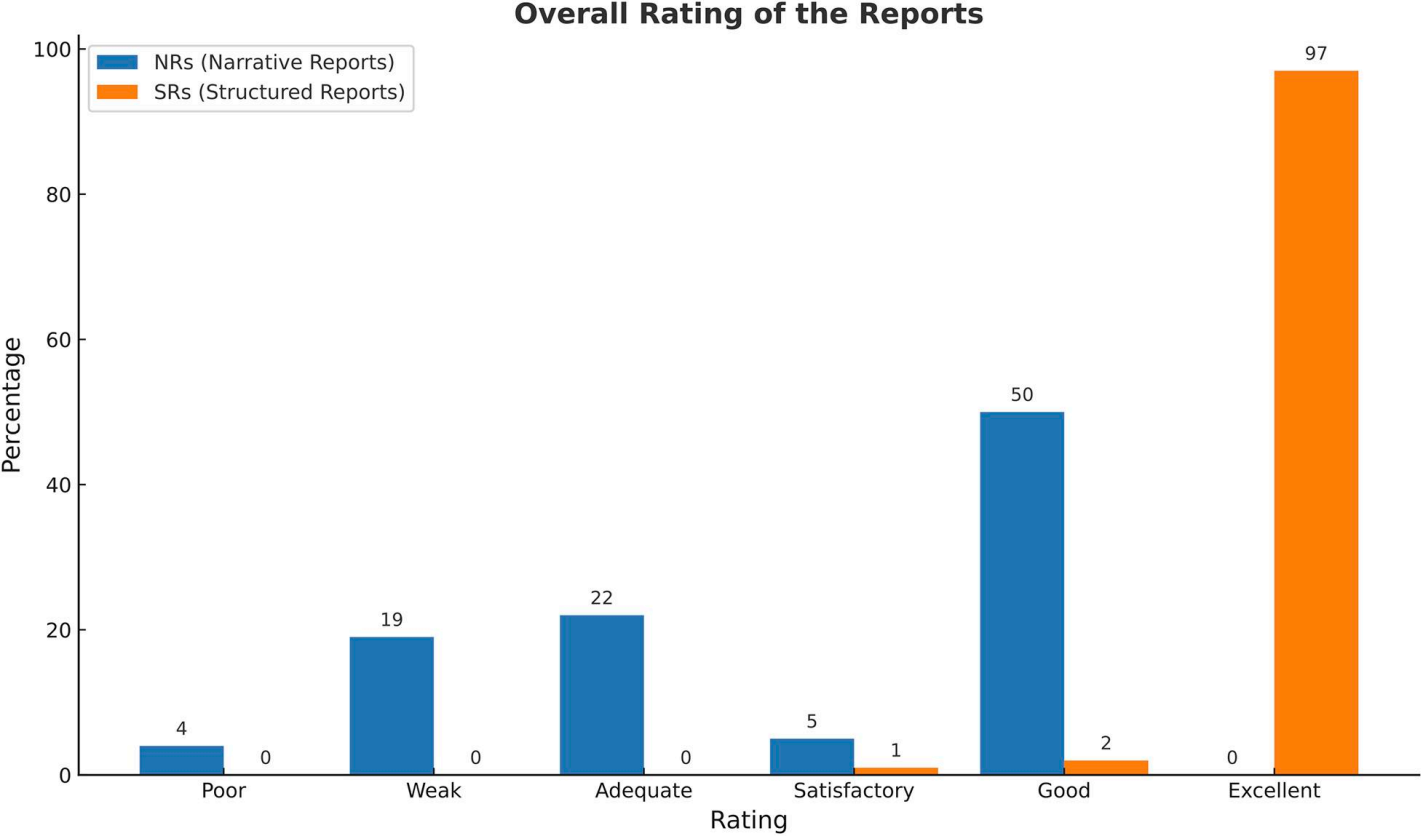
Overall rating of SR vs. NR.

Structured reports received predominantly excellent ratings (97%), with minimal variability. Narrative reports were more heterogeneous, with a mean score of 3.77 compared to 5.96 for SRs. Statistical analysis confirmed SRs were rated significantly higher overall (*p* < 0.001). The results are displayed in Figure 3. A summary of all results above can be seen in Table 1.

**Table 1:** Overall results.

Question	NR (median)	SR (median)	*p*
Was it possible to make a decision regarding the future therapeutic approach based on the report?	98%	99%	<0.05
Are the provided details sufficient für the proposed treatment?	100%	100%	>0.05
How easily could relevant information be extracted from the report?	72%	91%	<0.05
Are there missing key features?	91%	4%	<0.05
Was the documentation sufficiently detailed?	53%	98%	<0.05
Was the order and structure of findings appropriate and logical?	54%	100%	<0.05
How much trust do you place in the given information?	3.90	5.98	<0.05
How would you rate the comprehensibility of the report?	3.71	5.95	<0.05
How would you rate the report overall?	3.77	5.96	<0.05

### 3.11 Interrater agreement between dentists

**Figure 4:**
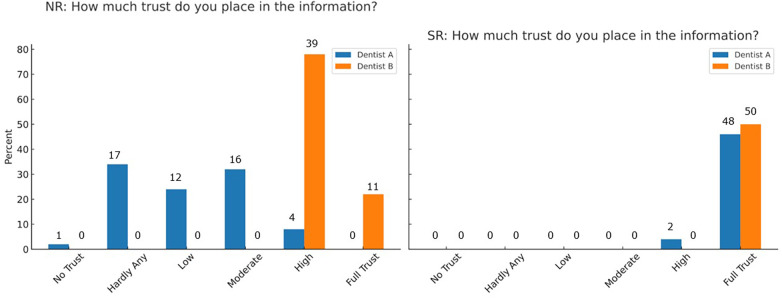
Bar chart comparing the questionnaire responses of the two dentists regarding the narrative reports (left) and the structured reports (right).

Interrater reliability was moderate for narrative reports (Cohen's *κ* = 0.380; Krippendorff's *α* = 0.366) and nearly perfect for structured reports (Cohen's *κ* = 0.947; Krippendorff's *α* = 0.947). Disagreement in NRs was especially evident in subjective areas such as trust and clarity. The results underscore the greater consistency and objectivity of structured reporting. Figure 4 shows the difference between the raters in both structured and narrative reports.

## 4 Discussion

### 4.1 Summary and interpretation of findings

This retrospective study evaluated the impact of structured reporting compared to traditional narrative reporting for dental radiographs using a standardized assessment across ten key dimensions. While both formats were deemed sufficient for therapeutic decision-making, structured reports consistently outperformed narrative reports in nearly all other areas, including report completeness, clarity, level of detail, and linguistic quality—findings that echo results from previous studies on structured reporting across different radiologic domains (11).

Structured reports were perceived as easier to read and interpret, with significantly higher ratings in information accessibility and diagnostic trust. These results mirror earlier evidence that a structured format can bolster clinicians’ confidence in the content and usability of radiologic documentation (23).

Moreover, interrater agreement was markedly higher for structured reports in our study. This finding underscores a major strength of structured reporting, especially its ability to reduce subjective variability and promote consistency across practitioners. Similar improvements in inter-reader reliability and the standardized documentation of key features have been reported in abdominal imaging studies using structured templates (24).

The highly similar clinical and training backgrounds of the two expert evaluators offered a unique perspective on the duality of documentation quality. While this common ground led to a near-perfect consensus on the high quality and completeness of the structured reports, it conversely highlighted the severe variability and inconsistency in the narrative reports. This suggests that the lack of a standardized structure forces even experienced clinicians into subjective interpretation and occasional omissions—a variability that the structured format effectively eliminates. Notably, referring physicians in prior studies have also reported greater satisfaction with structured reports due to their improved clarity, organization, and perceived completeness (25).

### 4.2 Structured reporting in the dental context

While structured reporting has become increasingly established in general medical radiology, its application in dental radiology presents distinct opportunities and challenges. The dental field benefits from a narrower diagnostic focus, typically involving teeth, periodontal structures, and surrounding bone, which lends itself well to template-driven approaches.

However, the diversity of dental imaging modalities complicates the development of a universal template. The range spans from 2D imaging (e.g., periapical, bitewing, panoramic radiographs) to 3D techniques like cone beam computed tomography (CBCT), each requiring different types of data representation (4, 8, 26–29). Templates that are too rigid may hinder clinical flexibility, while overly loose structures risk losing the benefits of standardization.

Terminology is another key consideration. Whereas RadLex has been widely adopted in general radiology, SNODENT (Systematized Nomenclature of Dentistry) may offer a more suitable foundation for standardizing dental reports. Consistent language across systems is essential for improving communication, data interoperability, and integration into broader healthcare infrastructures (26).

Existing research has also demonstrated that structured reporting may contribute to improved clinical decision-making and long-term treatment outcomes. For instance, a study at Goethe University Frankfurt suggested that SR could aid in the evaluation of indirect restorations by standardizing diagnostic input, potentially impacting therapeutic longevity (30).

### 4.3 Results

The findings of this study underscore the considerable potential of structured reporting in dental radiology. While both narrative and structured reports proved adequate for supporting therapeutic decisions, structured reports outperformed narrative ones in nearly all other evaluated aspects. The results were in line with numerous published studies beyond dental imaging (18–22).

The most prominent difference between SR and NR was observed in the completeness of the reports. In particular, critical clinical information such as the documentation of restorations, periodontal findings, and bone changes was frequently missing in narrative reports. This aligns with findings from other radiological disciplines, where structured templates have been shown to improve thoroughness and systematic data capture. The process of extracting relevant information was also rated significantly more efficient for structured reports. Their standardized format enables faster identification of key findings, being an important advantage in clinical environments where time is limited and interdisciplinary communication is essential. Additionally, the linguistic quality of structured reports received consistently higher ratings. This is likely due to their consistent terminology and clearly organized phrasing, which greatly enhances clarity. Structured reports also scored significantly higher in terms of trust, a crucial factor for clinical confidence and decision-making. Another important finding concerns interrater reliability. Whereas evaluations of narrative reports varied considerably between the two reviewers, the structured reports showed near-complete agreement. This suggests that standardization not only improves documentation but also facilitates more consistent interpretation between clinicians.

Nonetheless, the results do have some downsides as well. In more complex or atypical cases, rigid templates may restrict diagnostic flexibility. The key challenge lies in designing reporting tools that strike a balance between structure and adaptability offering guidance without oversimplifying clinical reasoning. Furthermore, practical considerations must also be taken into account. The successful implementation of structured reporting systems depends on appropriate software, user training, and integration into existing workflows. Without this infrastructure, even well-designed systems may face resistance, particularly in small practices with limited resources. Beyond immediate clinical benefits, structured reporting holds promise for the future of diagnostic processes, especially in conjunction with artificial intelligence. Structured, machine-readable data provides a valuable foundation for automated analysis, algorithm training, and large-scale research.

### 4.4 Limitations

Despite its contributions and its clear results, this study has several limitations that must be considered.

The sample size was relatively small (*n* = 50) and drawn from a single institution. While suitable for initial insights, a larger and multicenter design would allow for greater statistical power and generalizability. Besides that, only periapical radiographs were evaluated, limiting the applicability of the findings to other dental imaging modalities. As diagnostic requirements differ across modalities, future studies should examine SR performance in panoramic, bitewing, and CBCT imaging (3). A potential limitation is that the similar clinical and training backgrounds of the evaluators, while beneficial for ensuring high-quality assessment standards, might not fully reflect the variability found in a larger, international, multi-specialty setting. However, this homogeneity was methodologically useful, serving as a control measure that ensured the differences found between the two report formats were solely attributable to the documentation structure. Furthermore, SRs were generated retrospectively by a single dentist using a self-developed template, whereas NRs were written in routine clinical contexts by three different practitioners. This discrepancy may have introduced variation not solely attributable to the report format itself. Additionally, the SR template was not externally validated or pilot-tested. There also must be acknowledged that the study did not measure time expenditure for each reporting method. Yet, reporting time is a relevant omission, as efficiency is a critical factor in daily clinical workflows. Likewise, since SRs and NRs were not produced by the same individuals under the same conditions, direct intra-person comparisons were not possible. Moreover, only one reporting platform (*Smart Radiology*) was used, which limits the generalizability to other SR systems. Different platforms may vary in usability, terminology, and customization and therefore lead to different outcomes. Finally, the study focused on perceived report quality but did not assess downstream clinical outcomes such as diagnostic accuracy or treatment success. Further outcome-based research will be necessary to evaluate the full clinical impact of SR. Future studies should also integrate time measurements to objectively quantify the efficiency gains provided by Structured Reporting, which our study only assessed subjectively. A further limitation is the lack of explicit stratification of the sample according to pathology type or complexity (e.g., grading of periodontal bone loss or size of periapical lesions). While the random selection of reports from routine practice aimed to reflect a general, unbiased clinical spectrum, future studies should investigate the specific benefits of Structured Reporting on documentation quality in high-complexity or subtle-finding cases to further generalize our findings. Another limitation stemming from the retrospective design is the lack of explicit quality control regarding the image acquisition technique, such as the consistent use of the paralleling technique. Although the radiographs were taken following the standard protocol of the collaborating practices, the study did not include a technical assessment of every image's quality. This introduces inherent variability, but since the same radiographic image was used for both the Narrative and Structured Report assessment, any influence of image quality was equally distributed across the two reporting formats, ensuring the validity of our comparative findings.

## 5 Conclusion

This study highlights the significant potential of structured reporting in dental radiology as compared to traditional narrative reporting. Structured reports demonstrated clear advantages in terms of completeness, level of detail, linguistic quality, logical structure, and clarity indicating a substantial improvement in overall reporting quality.

While both report types enabled reliable therapeutic decision-making, SRs were notably more standardized and consistent. However, further large-scale and long-term studies are needed to evaluate the broader clinical impact of SR implementation, including practitioner acceptance and influence on diagnostic accuracy and treatment planning. For less experienced clinicians, structured templates can act as procedural guides, ensuring thorough and standardized documentation.

In summary, structured reporting represents a forward-looking innovation in dental radiographic diagnostics. Its advantages in quality, standardization, and efficiency make it a valuable tool for modern dental care. Broader clinical implementation should be actively pursued, supported by further research into technical feasibility, user acceptance, and integration with AI and digital health systems to enhance diagnostic workflows and patient outcomes.

## Data Availability

The raw data supporting the conclusions of this article will be made available by the authors, without undue reservation.
